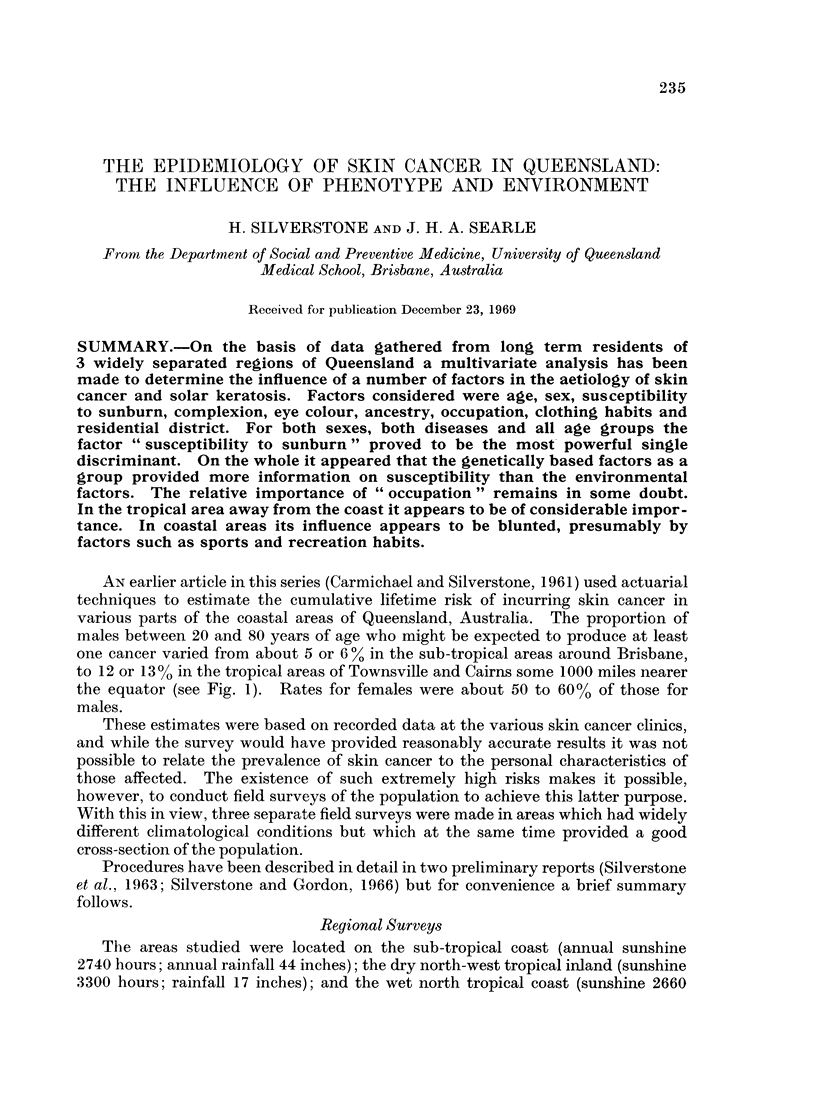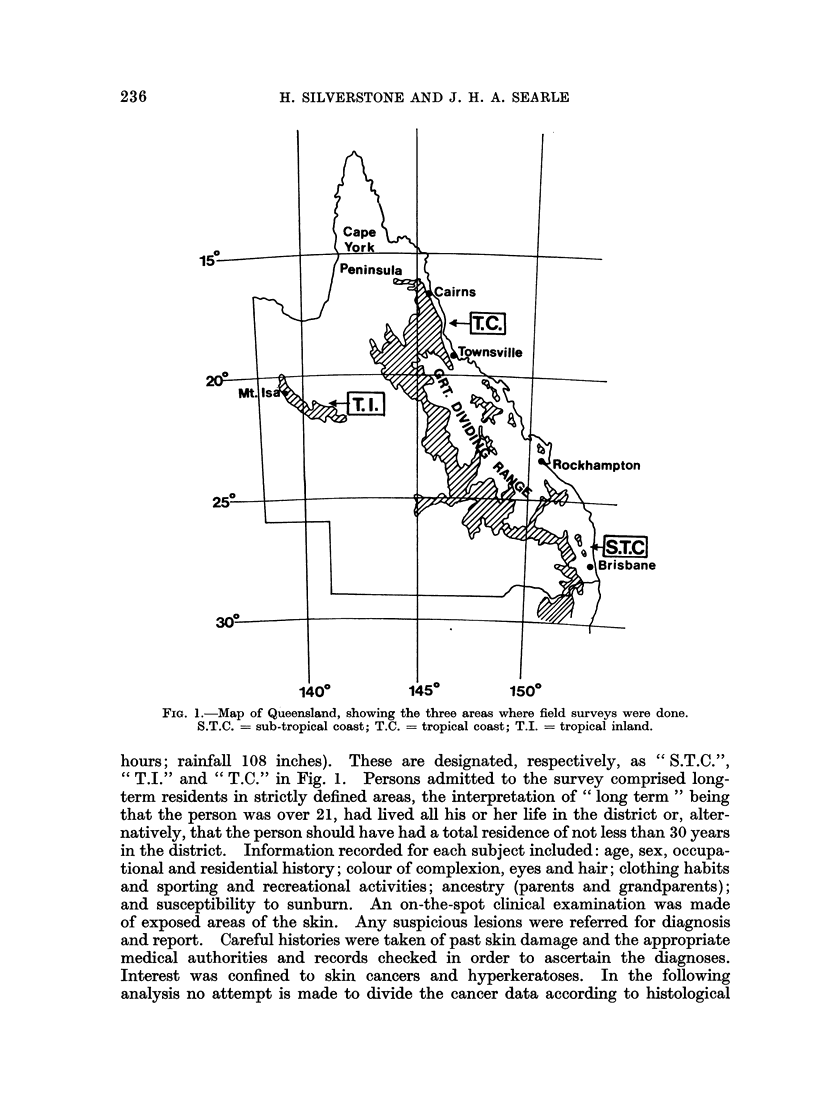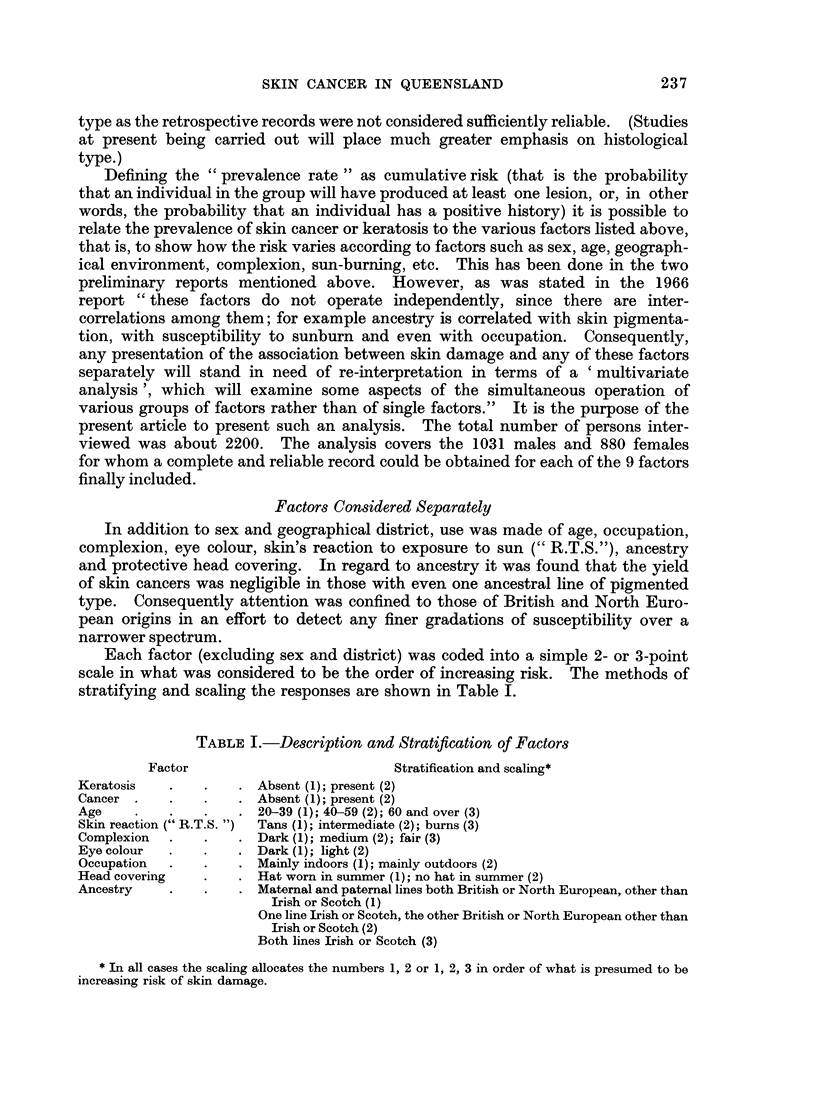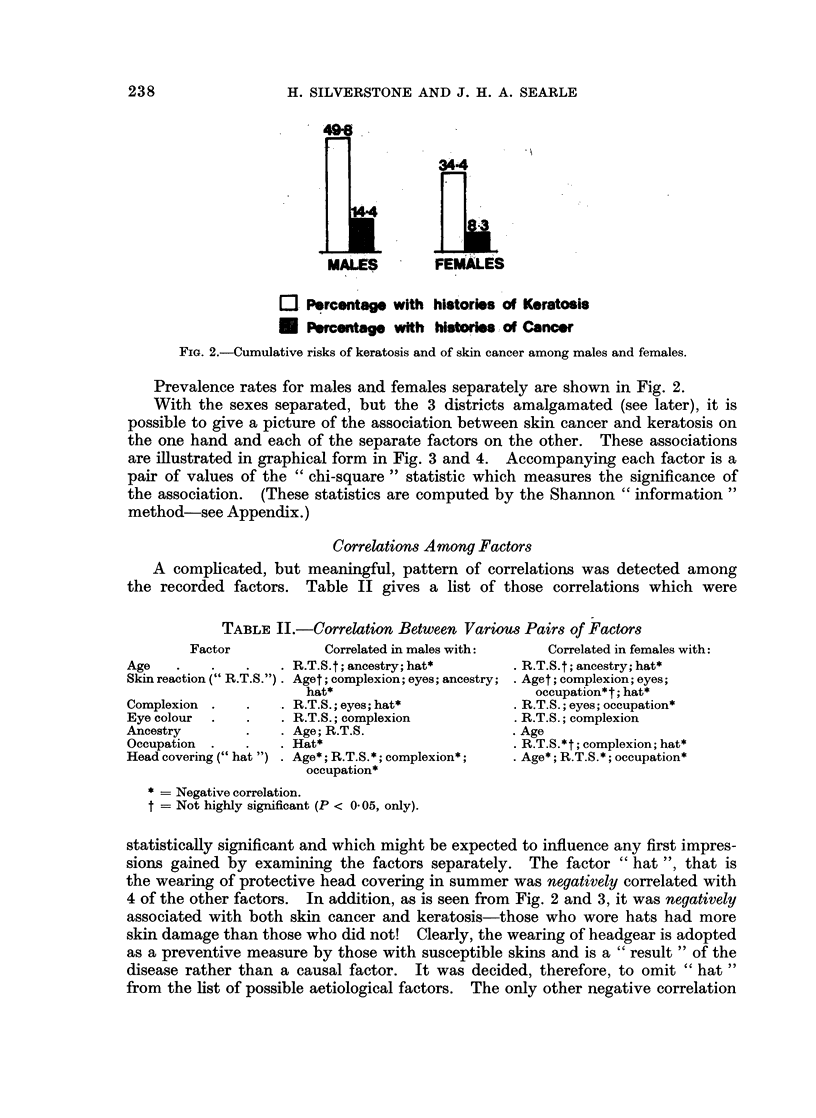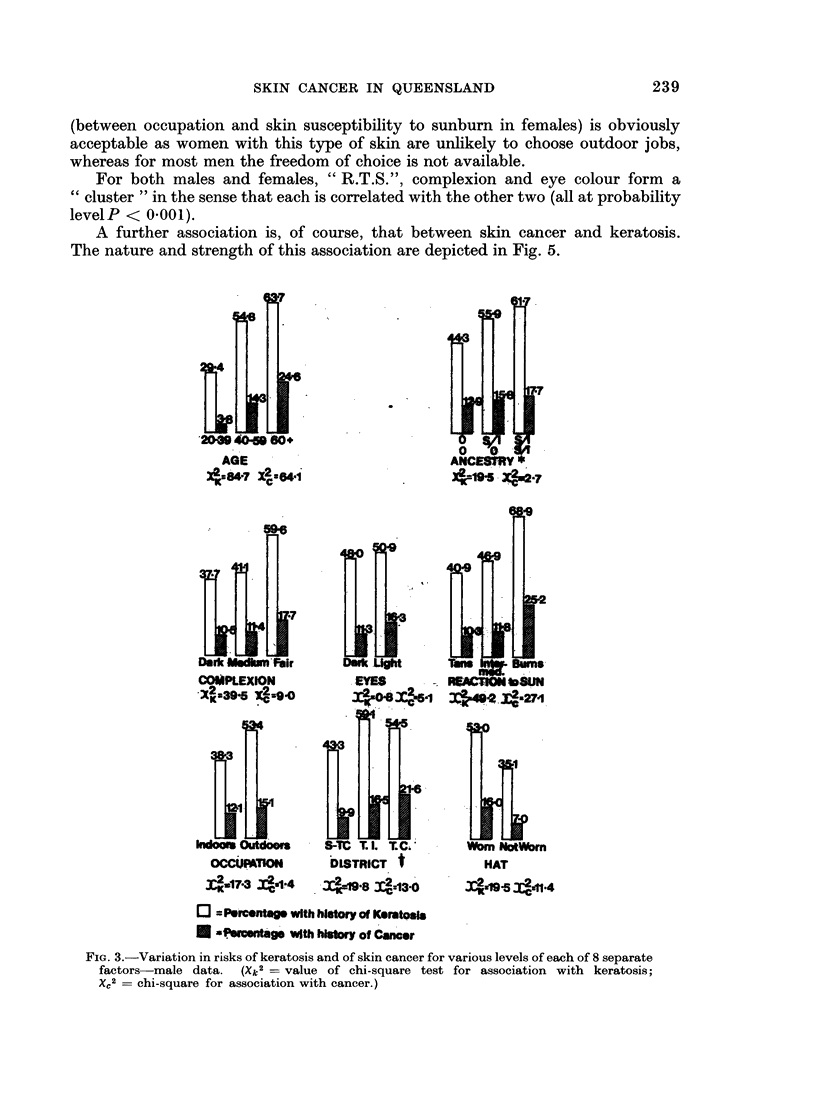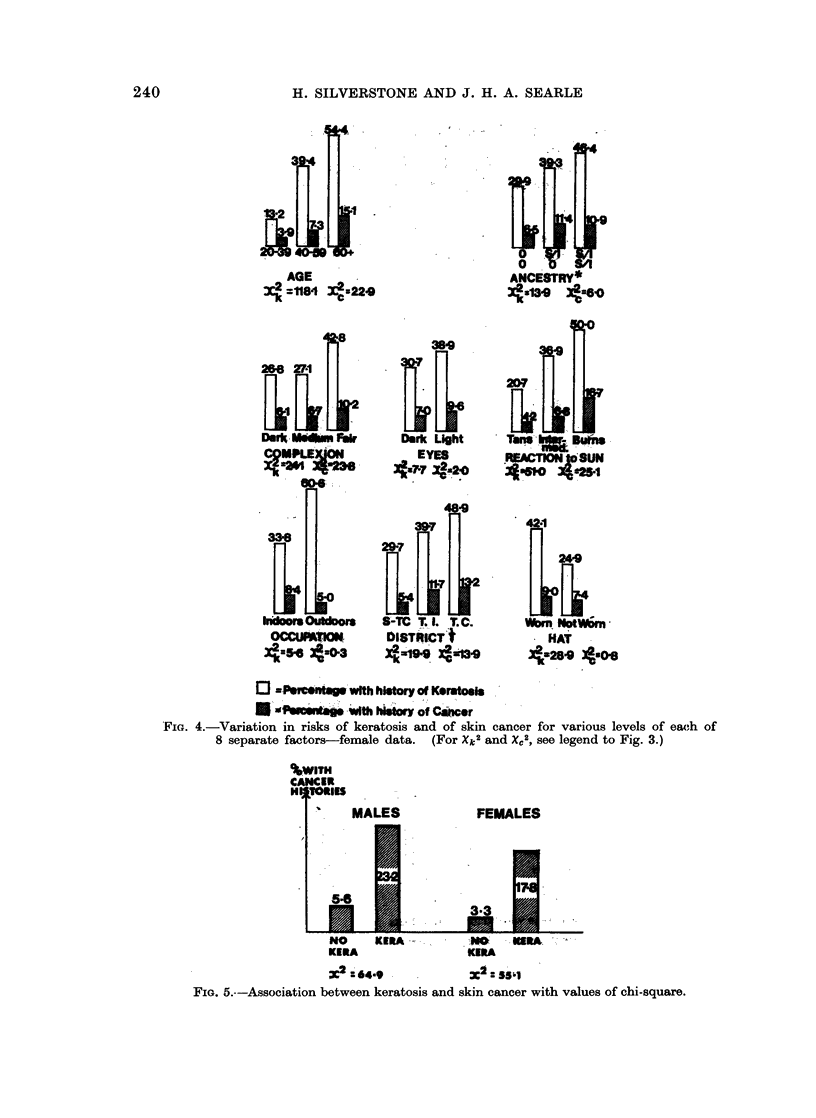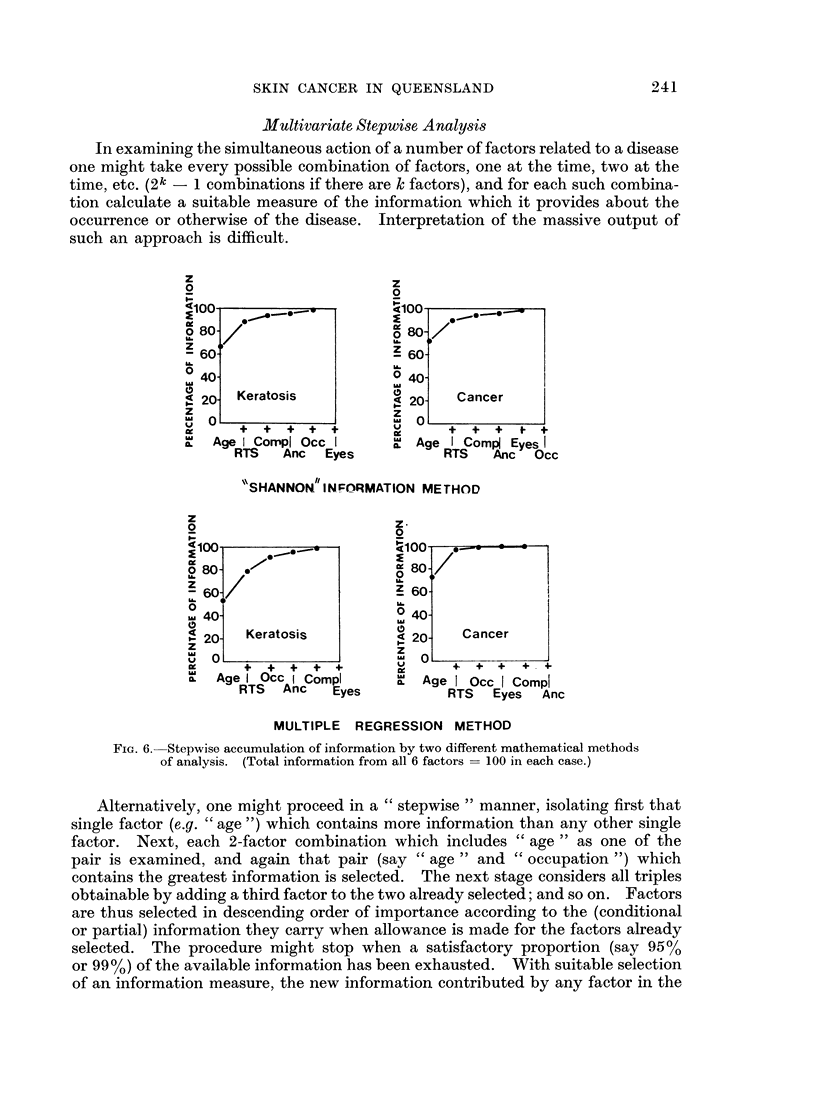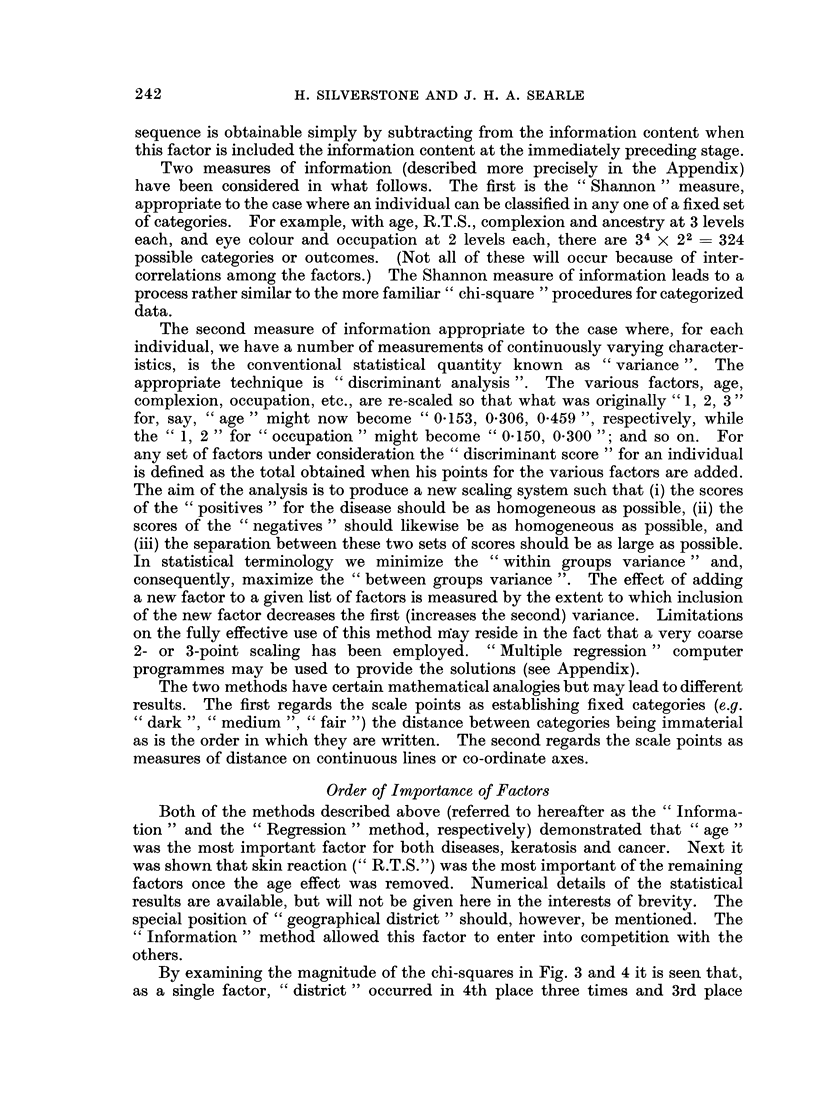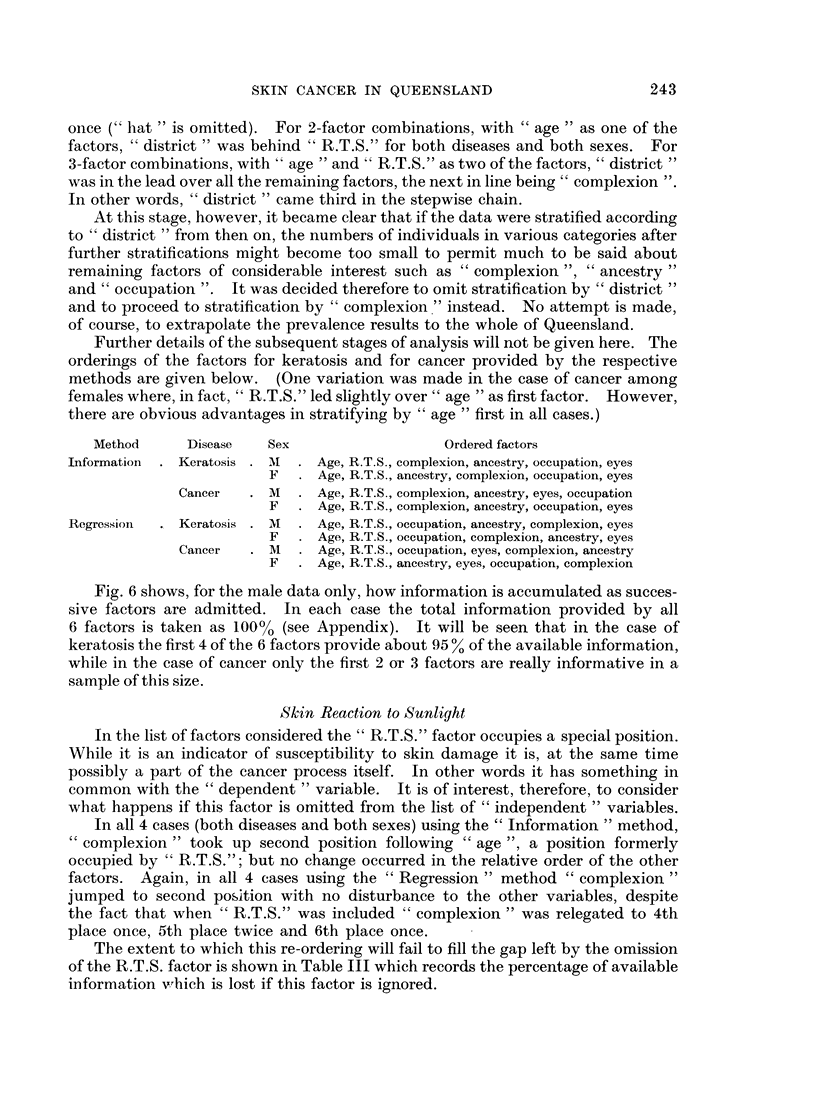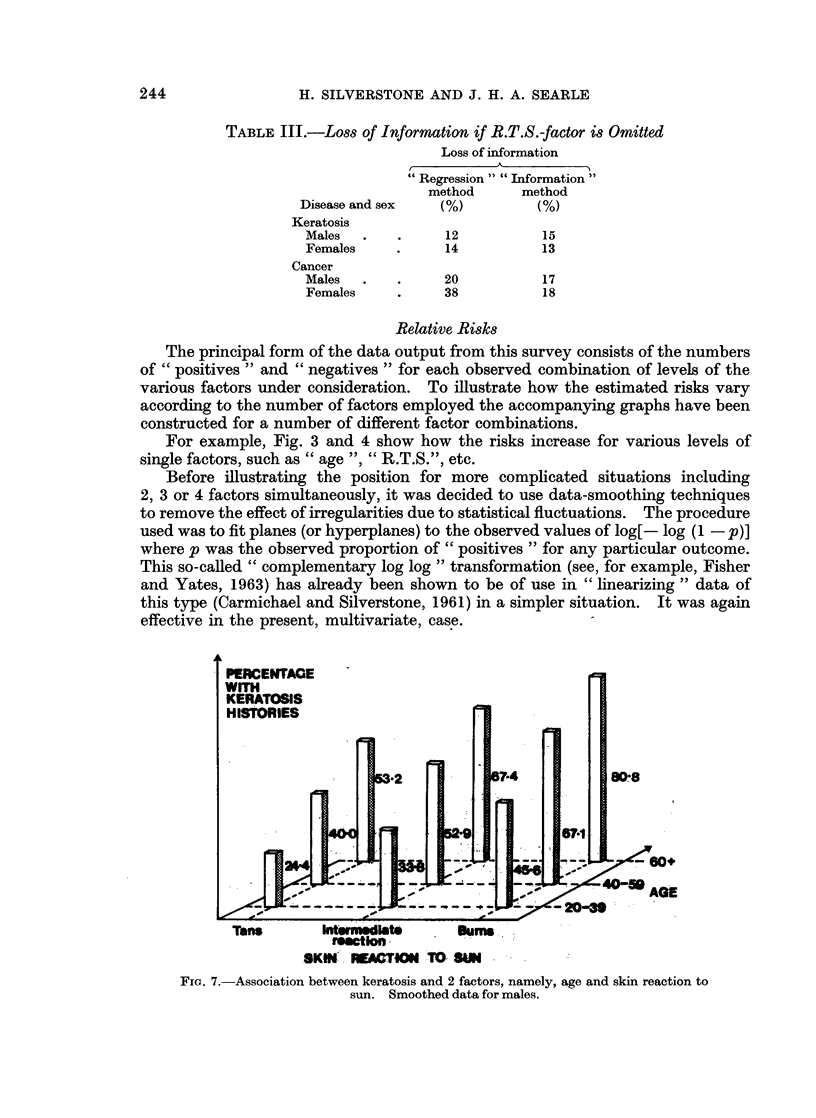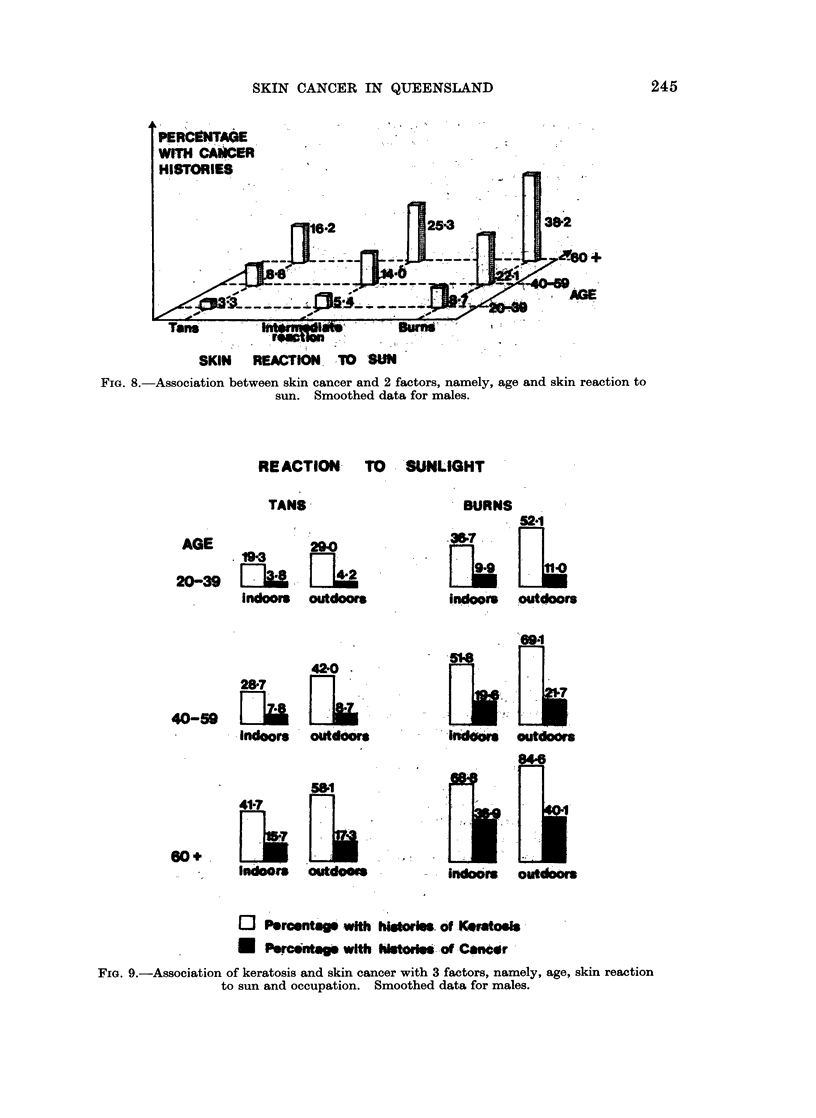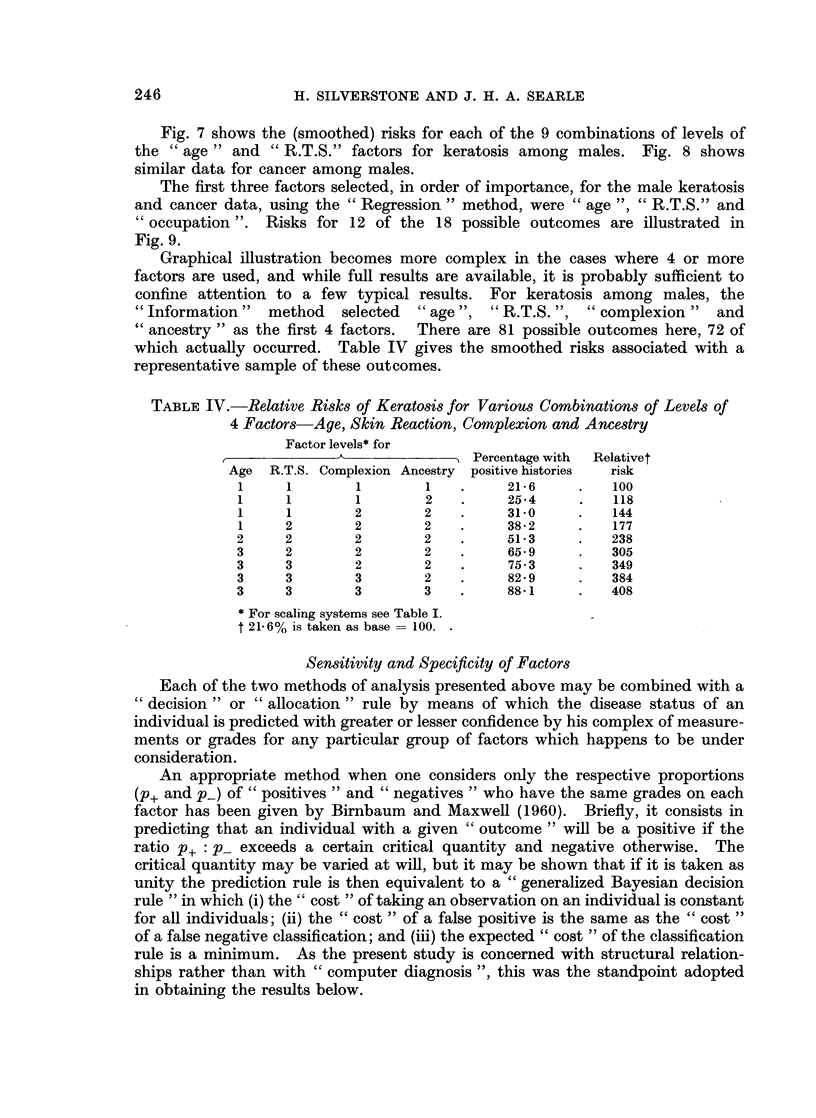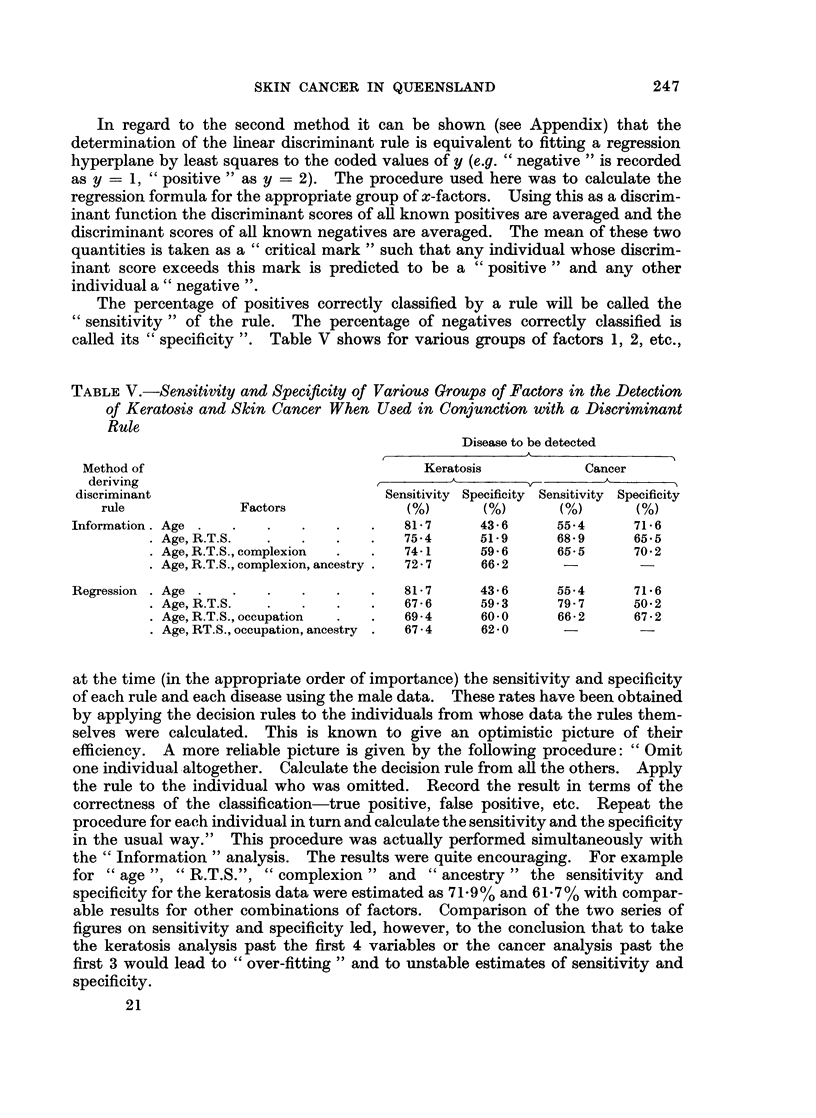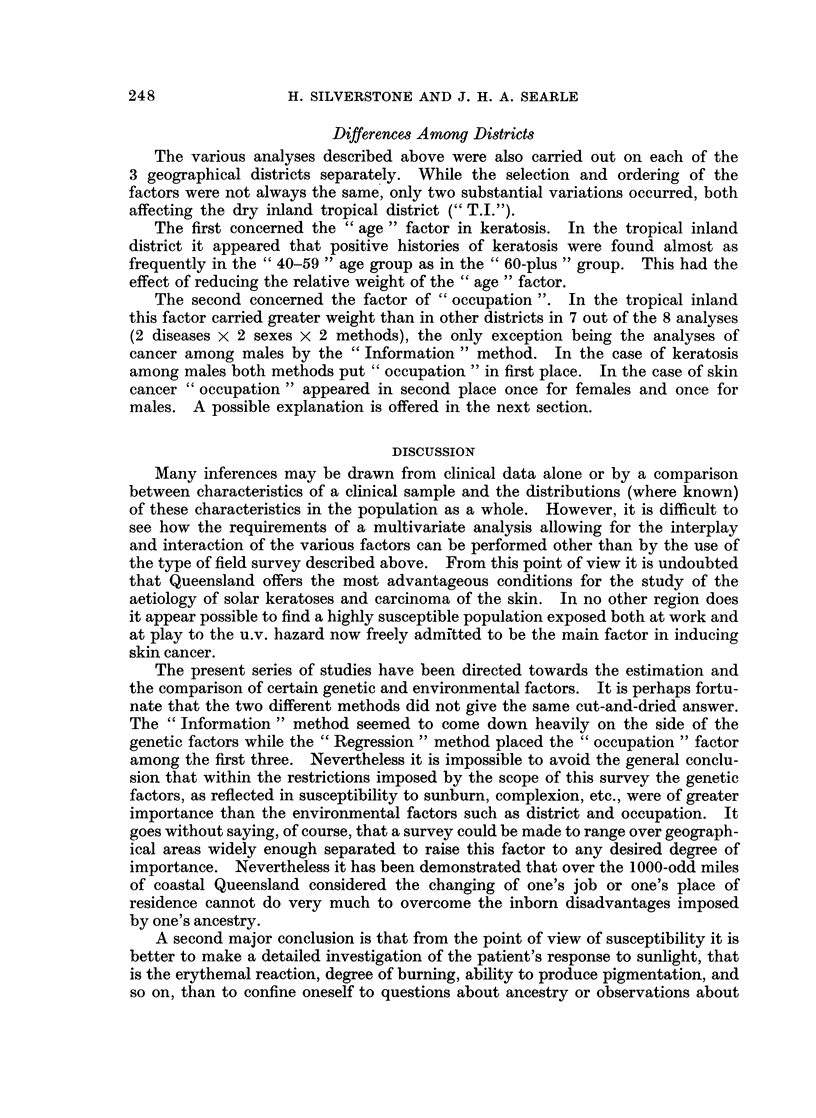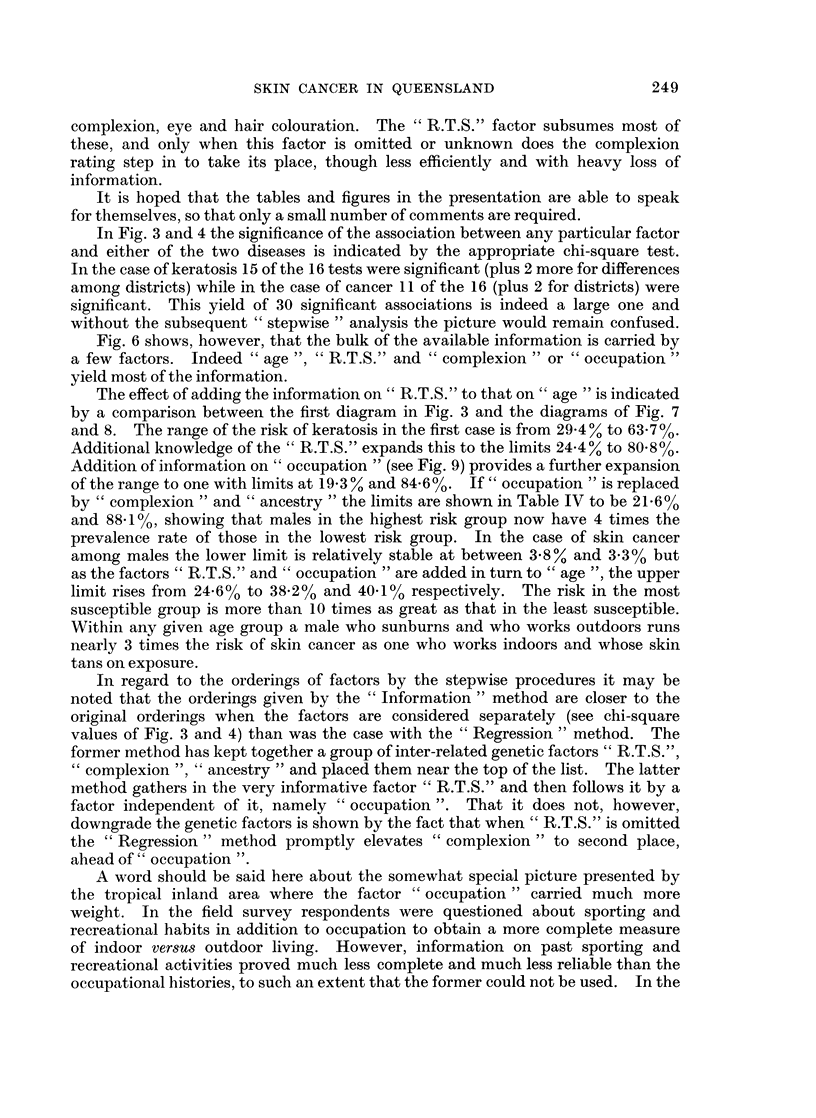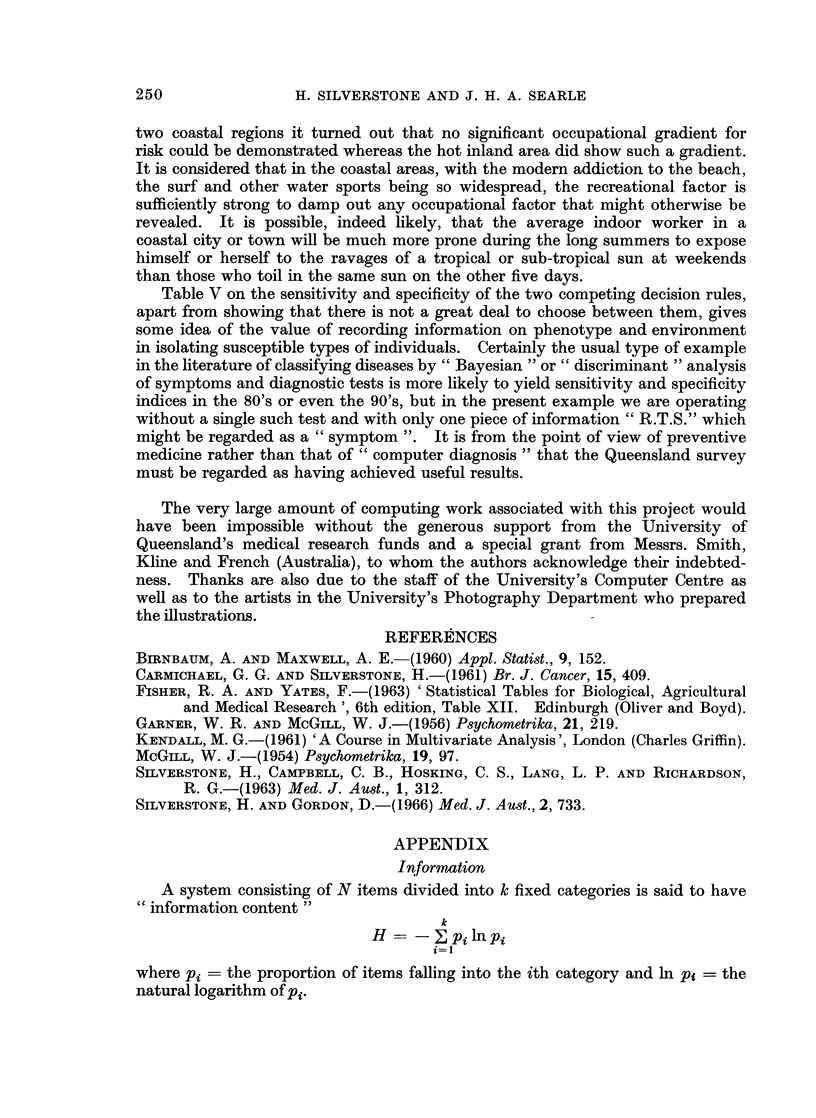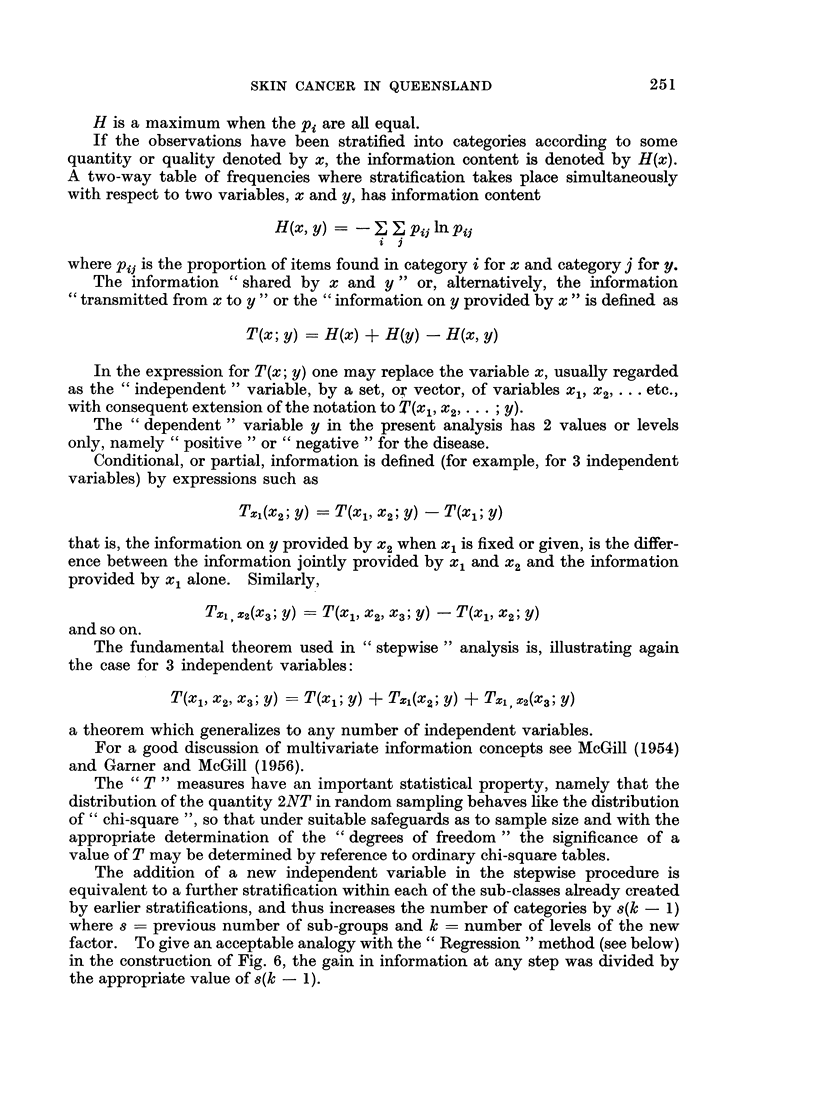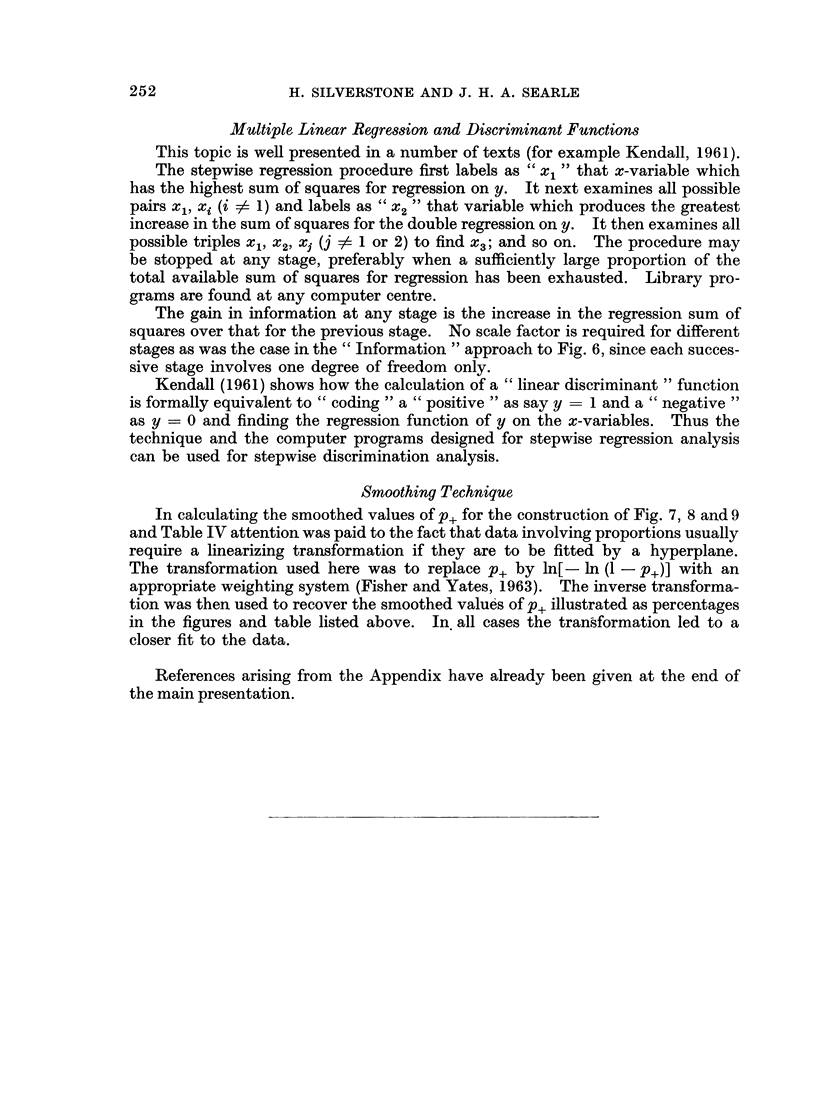# The Epidemiology of Skin Cancer in Queensland: The Influence of Phenotype and Environment

**DOI:** 10.1038/bjc.1970.27

**Published:** 1970-06

**Authors:** H. Silverstone, J. H. A. Searle

## Abstract

On the basis of data gathered from long term residents of 3 widely separated regions of Queensland a multivariate analysis has been made to determine the influence of a number of factors in the aetiology of skin cancer and solar keratosis. Factors considered were age, sex, susceptibility to sunburn, complexion, eye colour, ancestry, occupation, clothing habits and residential district. For both sexes, both diseases and all age groups the factor “susceptibility to sunburn” proved to be the most powerful single discriminant. On the whole it appeared that the genetically based factors as a group provided more information on susceptibility than the environmental factors. The relative importance of “occupation” remains in some doubt. In the tropical area away from the coast it appears to be of considerable importance. In coastal areas its influence appears to be blunted, presumably by factors such as sports and recreation habits.


### THE EPIDEMIOLOGY OF SKIN CANCER IN QUEENSLAND: THE INFLUENCE OF PHENOTYPE AND ENVIRONMENT

H. SILVERSTONE AND J. H. A. SEARLE

*From the Department of Social and Preventive Medicine, University of Queensland Medical School, Brisbane, Australia*

Received for publication December 23, 1969

**SUMMARY.—On the basis of data gathered from long term residents of 3 widely separated regions of Queensland a multivariate analysis has been made to determine the influence of a number of factors in the aetiology of skin cancer and solar keratosis. Factors considered were age, sex, susceptibility to sunburn, complexion, eye colour, ancestry, occupation, clothing habits and residential district. For both sexes, both diseases and all age groups the factor " susceptibility to sunburn " proved to be the most powerful single discriminant. On the whole it appeared that the genetically based factors as a group provided more information on susceptibility than the environmental factors. The relative importance of " occupation " remains in some doubt. In the tropical area away from the coast it appears to be of considerable importance. In coastal areas its influence appears to be blunted, presumably by factors such as sports and recreation habits.**

AN earlier article in this series (Carmichael and Silverstone, 1961) used actuarial techniques to estimate the cumulative lifetime risk of incurring skin cancer in various parts of the coastal areas of Queensland, Australia. The proportion of males between 20 and 80 years of age who might be expected to produce at least one cancer varied from about 5 or 6% in the sub-tropical areas around Brisbane, to 12 or 13% in the tropical areas of Townsville and Cairns some 1000 miles nearer the equator (see Fig. 1). Rates for females were about 50 to 60% of those for males.

These estimates were based on recorded data at the various skin cancer clinics, and while the survey would have provided reasonably accurate results it was not possible to relate the prevalence of skin cancer to the personal characteristics of those affected. The existence of such extremely high risks makes it possible, however, to conduct field surveys of the population to achieve this latter purpose. With this in view, three separate field surveys were made in areas which had widely different climatological conditions but which at the same time provided a good cross-section of the population.

Procedures have been described in detail in two preliminary reports (Silverstone *et al.*, 1963; Silverstone and Gordon, 1966) but for convenience a brief summary follows.

*Regional Surveys*

The areas studied were located on the sub-tropical coast (annual sunshine 2740 hours; annual rainfall 44 inches); the dry north-west tropical inland (sunshine 3300 hours; rainfall 17 inches); and the wet north tropical coast (sunshine 2660

FIG. 1.—Map of Queensland, showing the three areas where field surveys were done. S.T.C. = sub-tropical coast; T.C. = tropical coast; T.I. = tropical inland.

hours; rainfall 108 inches). These are designated, respectively, as " S.T.C.", " T.I." and " T.C." in Fig. 1. Persons admitted to the survey comprised long-term residents in strictly defined areas, the interpretation of " long term " being that the person was over 21, had lived all his or her life in the district or, alternatively, that the person should have had a total residence of not less than 30 years in the district. Information recorded for each subject included: age, sex, occupational and residential history; colour of complexion, eyes and hair; clothing habits and sporting and recreational activities; ancestry (parents and grandparents); and susceptibility to sunburn. An on-the-spot clinical examination was made of exposed areas of the skin. Any suspicious lesions were referred for diagnosis and report. Careful histories were taken of past skin damage and the appropriate medical authorities and records checked in order to ascertain the diagnoses. Interest was confined to skin cancers and hyperkeratoses. In the following analysis no attempt is made to divide the cancer data according to histological type as the retrospective records were not considered sufficiently reliable. (Studies at present being carried out will place much greater emphasis on histological type.)

Defining the " prevalence rate " as cumulative risk (that is the probability that an individual in the group will have produced at least one lesion, or, in other words, the probability that an individual has a positive history) it is possible to relate the prevalence of skin cancer or keratosis to the various factors listed above, that is, to show how the risk varies according to factors such as sex, age, geographical environment, complexion, sun-burning, etc. This has been done in the two preliminary reports mentioned above. However, as was stated in the 1966 report " these factors do not operate independently, since there are inter-correlations among them; for example ancestry is correlated with skin pigmentation, with susceptibility to sunburn and even with occupation. Consequently, any presentation of the association between skin damage and any of these factors separately will stand in need of re-interpretation in terms of a ' multivariate analysis ', which will examine some aspects of the simultaneous operation of various groups of factors rather than of single factors." It is the purpose of the present article to present such an analysis. The total number of persons interviewed was about 2200. The analysis covers the 1031 males and 880 females for whom a complete and reliable record could be obtained for each of the 9 factors finally included.

*Factors Considered Separately*

In addition to sex and geographical district, use was made of age, occupation, complexion, eye colour, skin's reaction to exposure to sun (" R.T.S."), ancestry and protective head covering. In regard to ancestry it was found that the yield of skin cancers was negligible in those with even one ancestral line of pigmented type. Consequently attention was confined to those of British and North European origins in an effort to detect any finer gradations of susceptibility over a narrower spectrum.

Each factor (excluding sex and district) was coded into a simple 2- or 3-point scale in what was considered to be the order of increasing risk. The methods of stratifying and scaling the responses are shown in Table I.

TABLE I.—*Description and Stratification of Factors*

| Factor | Stratification and scaling* |
|---|---|
| Keratosis | Absent (1); present (2) |
| Cancer | Absent (1); present (2) |
| Age | 20–39 (1); 40–59 (2); 60 and over (3) |
| Skin reaction (" R.T.S. ") | Tans (1); intermediate (2); burns (3) |
| Complexion | Dark (1); medium (2); fair (3) |
| Eye colour | Dark (1); light (2) |
| Occupation | Mainly indoors (1); mainly outdoors (2) |
| Head covering | Hat worn in summer (1); no hat in summer (2) |
| Ancestry | Maternal and paternal lines both British or North European, other than Irish or Scotch (1) |
| | One line Irish or Scotch, the other British or North European other than Irish or Scotch (2) |
| | Both lines Irish or Scotch (3) |

* In all cases the scaling allocates the numbers 1, 2 or 1, 2, 3 in order of what is presumed to be increasing risk of skin damage.

FIG. 2.—Cumulative risks of keratosis and of skin cancer among males and females.

Prevalence rates for males and females separately are shown in Fig. 2.

With the sexes separated, but the 3 districts amalgamated (see later), it is possible to give a picture of the association between skin cancer and keratosis on the one hand and each of the separate factors on the other. These associations are illustrated in graphical form in Fig. 3 and 4. Accompanying each factor is a pair of values of the " chi-square " statistic which measures the significance of the association. (These statistics are computed by the Shannon " information " method—see Appendix.)

### *Correlations Among Factors*

A complicated, but meaningful, pattern of correlations was detected among the recorded factors. Table II gives a list of those correlations which were

TABLE II.—*Correlation Between Various Pairs of Factors*

| Factor | Correlated in males with: | Correlated in females with: |
|---|---|---|
| Age | R.T.S.†; ancestry; hat* | R.T.S.†; ancestry; hat* |
| Skin reaction (" R.T.S.") | Age†; complexion; eyes; ancestry; hat* | Age†; complexion; eyes; occupation*†; hat* |
| Complexion | R.T.S.; eyes; hat* | R.T.S.; eyes; occupation* |
| Eye colour | R.T.S.; complexion | R.T.S.; complexion |
| Ancestry | Age; R.T.S. | Age |
| Occupation | Hat* | R.T.S.*†; complexion; hat* |
| Head covering (" hat ") | Age*; R.T.S.*; complexion*; occupation* | Age*; R.T.S.*; occupation* |

* = Negative correlation.
† = Not highly significant ($P < 0{\cdot}05$, only).

statistically significant and which might be expected to influence any first impressions gained by examining the factors separately. The factor " hat ", that is the wearing of protective head covering in summer was *negatively* correlated with 4 of the other factors. In addition, as is seen from Fig. 2 and 3, it was *negatively* associated with both skin cancer and keratosis—those who wore hats had more skin damage than those who did not! Clearly, the wearing of headgear is adopted as a preventive measure by those with susceptible skins and is a " result " of the disease rather than a causal factor. It was decided, therefore, to omit " hat " from the list of possible aetiological factors. The only other negative correlation

(between occupation and skin susceptibility to sunburn in females) is obviously acceptable as women with this type of skin are unlikely to choose outdoor jobs, whereas for most men the freedom of choice is not available.

For both males and females, " R.T.S.", complexion and eye colour form a " cluster " in the sense that each is correlated with the other two (all at probability level $P < 0{\cdot}001$).

A further association is, of course, that between skin cancer and keratosis. The nature and strength of this association are depicted in Fig. 5.

FIG. 3.—Variation in risks of keratosis and of skin cancer for various levels of each of 8 separate factors—male data. ($X_k^2$ = value of chi-square test for association with keratosis; $X_c^2$ = chi-square for association with cancer.)

FIG. 4.—Variation in risks of keratosis and of skin cancer for various levels of each of 8 separate factors—female data. (For $X_k^2$ and $X_c^2$, see legend to Fig. 3.)

FIG. 5.—Association between keratosis and skin cancer with values of chi-square.

*Multivariate Stepwise Analysis*

In examining the simultaneous action of a number of factors related to a disease one might take every possible combination of factors, one at the time, two at the time, etc. ($2^k - 1$ combinations if there are $k$ factors), and for each such combination calculate a suitable measure of the information which it provides about the occurrence or otherwise of the disease. Interpretation of the massive output of such an approach is difficult.

FIG. 6.—Stepwise accumulation of information by two different mathematical methods of analysis. (Total information from all 6 factors = 100 in each case.)

Alternatively, one might proceed in a " stepwise " manner, isolating first that single factor (*e.g.* " age ") which contains more information than any other single factor. Next, each 2-factor combination which includes " age " as one of the pair is examined, and again that pair (say " age " and " occupation ") which contains the greatest information is selected. The next stage considers all triples obtainable by adding a third factor to the two already selected; and so on. Factors are thus selected in descending order of importance according to the (conditional or partial) information they carry when allowance is made for the factors already selected. The procedure might stop when a satisfactory proportion (say 95% or 99%) of the available information has been exhausted. With suitable selection of an information measure, the new information contributed by any factor in the sequence is obtainable simply by subtracting from the information content when this factor is included the information content at the immediately preceding stage.

Two measures of information (described more precisely in the Appendix) have been considered in what follows. The first is the " Shannon " measure, appropriate to the case where an individual can be classified in any one of a fixed set of categories. For example, with age, R.T.S., complexion and ancestry at 3 levels each, and eye colour and occupation at 2 levels each, there are $3^4 \times 2^2 = 324$ possible categories or outcomes. (Not all of these will occur because of inter-correlations among the factors.) The Shannon measure of information leads to a process rather similar to the more familiar " chi-square " procedures for categorized data.

The second measure of information appropriate to the case where, for each individual, we have a number of measurements of continuously varying characteristics, is the conventional statistical quantity known as " variance ". The appropriate technique is " discriminant analysis ". The various factors, age, complexion, occupation, etc., are re-scaled so that what was originally " 1, 2, 3 " for, say, " age " might now become " 0·153, 0·306, 0·459 ", respectively, while the " 1, 2 " for " occupation " might become " 0·150, 0·300 "; and so on. For any set of factors under consideration the " discriminant score " for an individual is defined as the total obtained when his points for the various factors are added. The aim of the analysis is to produce a new scaling system such that (i) the scores of the " positives " for the disease should be as homogeneous as possible, (ii) the scores of the " negatives " should likewise be as homogeneous as possible, and (iii) the separation between these two sets of scores should be as large as possible. In statistical terminology we minimize the " within groups variance " and, consequently, maximize the " between groups variance ". The effect of adding a new factor to a given list of factors is measured by the extent to which inclusion of the new factor decreases the first (increases the second) variance. Limitations on the fully effective use of this method may reside in the fact that a very coarse 2- or 3-point scaling has been employed. " Multiple regression " computer programmes may be used to provide the solutions (see Appendix).

The two methods have certain mathematical analogies but may lead to different results. The first regards the scale points as establishing fixed categories (*e.g.* " dark ", " medium ", " fair ") the distance between categories being immaterial as is the order in which they are written. The second regards the scale points as measures of distance on continuous lines or co-ordinate axes.

*Order of Importance of Factors*

Both of the methods described above (referred to hereafter as the " Information " and the " Regression " method, respectively) demonstrated that " age " was the most important factor for both diseases, keratosis and cancer. Next it was shown that skin reaction (" R.T.S.") was the most important of the remaining factors once the age effect was removed. Numerical details of the statistical results are available, but will not be given here in the interests of brevity. The special position of " geographical district " should, however, be mentioned. The " Information " method allowed this factor to enter into competition with the others.

By examining the magnitude of the chi-squares in Fig. 3 and 4 it is seen that, as a single factor, " district " occurred in 4th place three times and 3rd place once (" hat " is omitted). For 2-factor combinations, with " age " as one of the factors, " district " was behind " R.T.S." for both diseases and both sexes. For 3-factor combinations, with " age " and " R.T.S." as two of the factors, " district " was in the lead over all the remaining factors, the next in line being " complexion ". In other words, " district " came third in the stepwise chain.

At this stage, however, it became clear that if the data were stratified according to " district " from then on, the numbers of individuals in various categories after further stratifications might become too small to permit much to be said about remaining factors of considerable interest such as " complexion ", " ancestry " and " occupation ". It was decided therefore to omit stratification by " district " and to proceed to stratification by " complexion " instead. No attempt is made, of course, to extrapolate the prevalence results to the whole of Queensland.

Further details of the subsequent stages of analysis will not be given here. The orderings of the factors for keratosis and for cancer provided by the respective methods are given below. (One variation was made in the case of cancer among females where, in fact, " R.T.S." led slightly over " age " as first factor. However, there are obvious advantages in stratifying by " age " first in all cases.)

| Method | Disease | Sex | Ordered factors |
|---|---|---|---|
| Information . | Keratosis . | M . | Age, R.T.S., complexion, ancestry, occupation, eyes |
| | | F . | Age, R.T.S., ancestry, complexion, occupation, eyes |
| | Cancer . | M . | Age, R.T.S., complexion, ancestry, eyes, occupation |
| | | F . | Age, R.T.S., complexion, ancestry, occupation, eyes |
| Regression . | Keratosis . | M . | Age, R.T.S., occupation, ancestry, complexion, eyes |
| | | F . | Age, R.T.S., occupation, complexion, ancestry, eyes |
| | Cancer . | M . | Age, R.T.S., occupation, eyes, complexion, ancestry |
| | | F . | Age, R.T.S., ancestry, eyes, occupation, complexion |

Fig. 6 shows, for the male data only, how information is accumulated as successive factors are admitted. In each case the total information provided by all 6 factors is taken as 100% (see Appendix). It will be seen that in the case of keratosis the first 4 of the 6 factors provide about 95% of the available information, while in the case of cancer only the first 2 or 3 factors are really informative in a sample of this size.

### *Skin Reaction to Sunlight*

In the list of factors considered the " R.T.S." factor occupies a special position. While it is an indicator of susceptibility to skin damage it is, at the same time possibly a part of the cancer process itself. In other words it has something in common with the " dependent " variable. It is of interest, therefore, to consider what happens if this factor is omitted from the list of " independent " variables.

In all 4 cases (both diseases and both sexes) using the " Information " method, " complexion " took up second position following " age ", a position formerly occupied by " R.T.S."; but no change occurred in the relative order of the other factors. Again, in all 4 cases using the " Regression " method " complexion " jumped to second position with no disturbance to the other variables, despite the fact that when " R.T.S." was included " complexion " was relegated to 4th place once, 5th place twice and 6th place once.

The extent to which this re-ordering will fail to fill the gap left by the omission of the R.T.S. factor is shown in Table III which records the percentage of available information which is lost if this factor is ignored.

TABLE III.—*Loss of Information if R.T.S.-factor is Omitted*

| Disease and sex | Loss of information: " Regression " method (%) | Loss of information: " Information " method (%) |
|---|---|---|
| Keratosis | | |
| Males | 12 | 15 |
| Females | 14 | 13 |
| Cancer | | |
| Males | 20 | 17 |
| Females | 38 | 18 |

*Relative Risks*

The principal form of the data output from this survey consists of the numbers of " positives " and " negatives " for each observed combination of levels of the various factors under consideration. To illustrate how the estimated risks vary according to the number of factors employed the accompanying graphs have been constructed for a number of different factor combinations.

For example, Fig. 3 and 4 show how the risks increase for various levels of single factors, such as " age ", " R.T.S.", etc.

Before illustrating the position for more complicated situations including 2, 3 or 4 factors simultaneously, it was decided to use data-smoothing techniques to remove the effect of irregularities due to statistical fluctuations. The procedure used was to fit planes (or hyperplanes) to the observed values of $\log[-\log(1-p)]$ where $p$ was the observed proportion of " positives " for any particular outcome. This so-called " complementary log log " transformation (see, for example, Fisher and Yates, 1963) has already been shown to be of use in " linearizing " data of this type (Carmichael and Silverstone, 1961) in a simpler situation. It was again effective in the present, multivariate, case.

FIG. 7.—Association between keratosis and 2 factors, namely, age and skin reaction to sun. Smoothed data for males.

FIG. 8.—Association between skin cancer and 2 factors, namely, age and skin reaction to sun. Smoothed data for males.

FIG. 9.—Association of keratosis and skin cancer with 3 factors, namely, age, skin reaction to sun and occupation. Smoothed data for males.

Fig. 7 shows the (smoothed) risks for each of the 9 combinations of levels of the " age " and " R.T.S." factors for keratosis among males. Fig. 8 shows similar data for cancer among males.

The first three factors selected, in order of importance, for the male keratosis and cancer data, using the " Regression " method, were " age ", " R.T.S." and " occupation ". Risks for 12 of the 18 possible outcomes are illustrated in Fig. 9.

Graphical illustration becomes more complex in the cases where 4 or more factors are used, and while full results are available, it is probably sufficient to confine attention to a few typical results. For keratosis among males, the " Information " method selected " age ", " R.T.S. ", " complexion " and " ancestry " as the first 4 factors. There are 81 possible outcomes here, 72 of which actually occurred. Table IV gives the smoothed risks associated with a representative sample of these outcomes.

TABLE IV.—*Relative Risks of Keratosis for Various Combinations of Levels of 4 Factors—Age, Skin Reaction, Complexion and Ancestry*

| Factor levels* for | | | | Percentage with | Relative† |
|---|---|---|---|---|---|
| Age | R.T.S. | Complexion | Ancestry | positive histories | risk |
| 1 | 1 | 1 | 1 | 21·6 | 100 |
| 1 | 1 | 1 | 2 | 25·4 | 118 |
| 1 | 1 | 2 | 2 | 31·0 | 144 |
| 1 | 2 | 2 | 2 | 38·2 | 177 |
| 2 | 2 | 2 | 2 | 51·3 | 238 |
| 3 | 2 | 2 | 2 | 65·9 | 305 |
| 3 | 3 | 2 | 2 | 75·3 | 349 |
| 3 | 3 | 3 | 2 | 82·9 | 384 |
| 3 | 3 | 3 | 3 | 88·1 | 408 |

* For scaling systems see Table I.
† 21·6% is taken as base = 100.

*Sensitivity and Specificity of Factors*

Each of the two methods of analysis presented above may be combined with a " decision " or " allocation " rule by means of which the disease status of an individual is predicted with greater or lesser confidence by his complex of measurements or grades for any particular group of factors which happens to be under consideration.

An appropriate method when one considers only the respective proportions ($p_+$ and $p_-$) of " positives " and " negatives " who have the same grades on each factor has been given by Birnbaum and Maxwell (1960). Briefly, it consists in predicting that an individual with a given " outcome " will be a positive if the ratio $p_+ : p_-$ exceeds a certain critical quantity and negative otherwise. The critical quantity may be varied at will, but it may be shown that if it is taken as unity the prediction rule is then equivalent to a " generalized Bayesian decision rule " in which (i) the " cost " of taking an observation on an individual is constant for all individuals; (ii) the " cost " of a false positive is the same as the " cost " of a false negative classification; and (iii) the expected " cost " of the classification rule is a minimum. As the present study is concerned with structural relationships rather than with " computer diagnosis ", this was the standpoint adopted in obtaining the results below.

In regard to the second method it can be shown (see Appendix) that the determination of the linear discriminant rule is equivalent to fitting a regression hyperplane by least squares to the coded values of $y$ (*e.g.* " negative " is recorded as $y = 1$, " positive " as $y = 2$). The procedure used here was to calculate the regression formula for the appropriate group of $x$-factors. Using this as a discriminant function the discriminant scores of all known positives are averaged and the discriminant scores of all known negatives are averaged. The mean of these two quantities is taken as a " critical mark " such that any individual whose discriminant score exceeds this mark is predicted to be a " positive " and any other individual a " negative ".

The percentage of positives correctly classified by a rule will be called the " sensitivity " of the rule. The percentage of negatives correctly classified is called its " specificity ". Table V shows for various groups of factors 1, 2, etc.,

TABLE V.—*Sensitivity and Specificity of Various Groups of Factors in the Detection of Keratosis and Skin Cancer When Used in Conjunction with a Discriminant Rule*

| | | Disease to be detected | | | |
|---|---|---|---|---|---|
| Method of deriving discriminant rule | Factors | Keratosis | | Cancer | |
| | | Sensitivity (%) | Specificity (%) | Sensitivity (%) | Specificity (%) |
| Information | Age | 81·7 | 43·6 | 55·4 | 71·6 |
| | Age, R.T.S. | 75·4 | 51·9 | 68·9 | 65·5 |
| | Age, R.T.S., complexion | 74·1 | 59·6 | 65·5 | 70·2 |
| | Age, R.T.S., complexion, ancestry | 72·7 | 66·2 | — | — |
| Regression | Age | 81·7 | 43·6 | 55·4 | 71·6 |
| | Age, R.T.S. | 67·6 | 59·3 | 79·7 | 50·2 |
| | Age, R.T.S., occupation | 69·4 | 60·0 | 66·2 | 67·2 |
| | Age, RT.S., occupation, ancestry | 67·4 | 62·0 | — | — |

at the time (in the appropriate order of importance) the sensitivity and specificity of each rule and each disease using the male data. These rates have been obtained by applying the decision rules to the individuals from whose data the rules themselves were calculated. This is known to give an optimistic picture of their efficiency. A more reliable picture is given by the following procedure: " Omit one individual altogether. Calculate the decision rule from all the others. Apply the rule to the individual who was omitted. Record the result in terms of the correctness of the classification—true positive, false positive, etc. Repeat the procedure for each individual in turn and calculate the sensitivity and the specificity in the usual way." This procedure was actually performed simultaneously with the " Information " analysis. The results were quite encouraging. For example for " age ", " R.T.S.", " complexion " and " ancestry " the sensitivity and specificity for the keratosis data were estimated as 71·9% and 61·7% with comparable results for other combinations of factors. Comparison of the two series of figures on sensitivity and specificity led, however, to the conclusion that to take the keratosis analysis past the first 4 variables or the cancer analysis past the first 3 would lead to " over-fitting " and to unstable estimates of sensitivity and specificity.

### *Differences Among Districts*

The various analyses described above were also carried out on each of the 3 geographical districts separately. While the selection and ordering of the factors were not always the same, only two substantial variations occurred, both affecting the dry inland tropical district (" T.I.").

The first concerned the " age " factor in keratosis. In the tropical inland district it appeared that positive histories of keratosis were found almost as frequently in the " 40–59 " age group as in the " 60-plus " group. This had the effect of reducing the relative weight of the " age " factor.

The second concerned the factor of " occupation ". In the tropical inland this factor carried greater weight than in other districts in 7 out of the 8 analyses (2 diseases × 2 sexes × 2 methods), the only exception being the analyses of cancer among males by the " Information " method. In the case of keratosis among males both methods put " occupation " in first place. In the case of skin cancer " occupation " appeared in second place once for females and once for males. A possible explanation is offered in the next section.

### DISCUSSION

Many inferences may be drawn from clinical data alone or by a comparison between characteristics of a clinical sample and the distributions (where known) of these characteristics in the population as a whole. However, it is difficult to see how the requirements of a multivariate analysis allowing for the interplay and interaction of the various factors can be performed other than by the use of the type of field survey described above. From this point of view it is undoubted that Queensland offers the most advantageous conditions for the study of the aetiology of solar keratoses and carcinoma of the skin. In no other region does it appear possible to find a highly susceptible population exposed both at work and at play to the u.v. hazard now freely admitted to be the main factor in inducing skin cancer.

The present series of studies have been directed towards the estimation and the comparison of certain genetic and environmental factors. It is perhaps fortunate that the two different methods did not give the same cut-and-dried answer. The " Information " method seemed to come down heavily on the side of the genetic factors while the " Regression " method placed the " occupation " factor among the first three. Nevertheless it is impossible to avoid the general conclusion that within the restrictions imposed by the scope of this survey the genetic factors, as reflected in susceptibility to sunburn, complexion, etc., were of greater importance than the environmental factors such as district and occupation. It goes without saying, of course, that a survey could be made to range over geographical areas widely enough separated to raise this factor to any desired degree of importance. Nevertheless it has been demonstrated that over the 1000-odd miles of coastal Queensland considered the changing of one's job or one's place of residence cannot do very much to overcome the inborn disadvantages imposed by one's ancestry.

A second major conclusion is that from the point of view of susceptibility it is better to make a detailed investigation of the patient's response to sunlight, that is the erythemal reaction, degree of burning, ability to produce pigmentation, and so on, than to confine oneself to questions about ancestry or observations about complexion, eye and hair colouration. The " R.T.S." factor subsumes most of these, and only when this factor is omitted or unknown does the complexion rating step in to take its place, though less efficiently and with heavy loss of information.

It is hoped that the tables and figures in the presentation are able to speak for themselves, so that only a small number of comments are required.

In Fig. 3 and 4 the significance of the association between any particular factor and either of the two diseases is indicated by the appropriate chi-square test. In the case of keratosis 15 of the 16 tests were significant (plus 2 more for differences among districts) while in the case of cancer 11 of the 16 (plus 2 for districts) were significant. This yield of 30 significant associations is indeed a large one and without the subsequent " stepwise " analysis the picture would remain confused.

Fig. 6 shows, however, that the bulk of the available information is carried by a few factors. Indeed " age ", " R.T.S." and " complexion " or " occupation " yield most of the information.

The effect of adding the information on " R.T.S." to that on " age " is indicated by a comparison between the first diagram in Fig. 3 and the diagrams of Fig. 7 and 8. The range of the risk of keratosis in the first case is from 29·4% to 63·7%. Additional knowledge of the " R.T.S." expands this to the limits 24·4% to 80·8%. Addition of information on " occupation " (see Fig. 9) provides a further expansion of the range to one with limits at 19·3% and 84·6%. If " occupation " is replaced by " complexion " and " ancestry " the limits are shown in Table IV to be 21·6% and 88·1%, showing that males in the highest risk group now have 4 times the prevalence rate of those in the lowest risk group. In the case of skin cancer among males the lower limit is relatively stable at between 3·8% and 3·3% but as the factors " R.T.S." and " occupation " are added in turn to " age ", the upper limit rises from 24·6% to 38·2% and 40·1% respectively. The risk in the most susceptible group is more than 10 times as great as that in the least susceptible. Within any given age group a male who sunburns and who works outdoors runs nearly 3 times the risk of skin cancer as one who works indoors and whose skin tans on exposure.

In regard to the orderings of factors by the stepwise procedures it may be noted that the orderings given by the " Information " method are closer to the original orderings when the factors are considered separately (see chi-square values of Fig. 3 and 4) than was the case with the " Regression " method. The former method has kept together a group of inter-related genetic factors " R.T.S.", " complexion ", " ancestry " and placed them near the top of the list. The latter method gathers in the very informative factor " R.T.S." and then follows it by a factor independent of it, namely " occupation ". That it does not, however, downgrade the genetic factors is shown by the fact that when " R.T.S." is omitted the " Regression " method promptly elevates " complexion " to second place, ahead of " occupation ".

A word should be said here about the somewhat special picture presented by the tropical inland area where the factor " occupation " carried much more weight. In the field survey respondents were questioned about sporting and recreational habits in addition to occupation to obtain a more complete measure of indoor *versus* outdoor living. However, information on past sporting and recreational activities proved much less complete and much less reliable than the occupational histories, to such an extent that the former could not be used. In the two coastal regions it turned out that no significant occupational gradient for risk could be demonstrated whereas the hot inland area did show such a gradient. It is considered that in the coastal areas, with the modern addiction to the beach, the surf and other water sports being so widespread, the recreational factor is sufficiently strong to damp out any occupational factor that might otherwise be revealed. It is possible, indeed likely, that the average indoor worker in a coastal city or town will be much more prone during the long summers to expose himself or herself to the ravages of a tropical or sub-tropical sun at weekends than those who toil in the same sun on the other five days.

Table V on the sensitivity and specificity of the two competing decision rules, apart from showing that there is not a great deal to choose between them, gives some idea of the value of recording information on phenotype and environment in isolating susceptible types of individuals. Certainly the usual type of example in the literature of classifying diseases by " Bayesian " or " discriminant " analysis of symptoms and diagnostic tests is more likely to yield sensitivity and specificity indices in the 80's or even the 90's, but in the present example we are operating without a single such test and with only one piece of information " R.T.S." which might be regarded as a " symptom ". It is from the point of view of preventive medicine rather than that of " computer diagnosis " that the Queensland survey must be regarded as having achieved useful results.

The very large amount of computing work associated with this project would have been impossible without the generous support from the University of Queensland's medical research funds and a special grant from Messrs. Smith, Kline and French (Australia), to whom the authors acknowledge their indebtedness. Thanks are also due to the staff of the University's Computer Centre as well as to the artists in the University's Photography Department who prepared the illustrations.

### REFERENCES

Birnbaum, A. and Maxwell, A. E.—(1960) *Appl. Statist.*, **9**, 152.
Carmichael, G. G. and Silverstone, H.—(1961) *Br. J. Cancer*, **15**, 409.
Fisher, R. A. and Yates, F.—(1963) 'Statistical Tables for Biological, Agricultural and Medical Research', 6th edition, Table XII. Edinburgh (Oliver and Boyd).
Garner, W. R. and McGill, W. J.—(1956) *Psychometrika*, **21**, 219.
Kendall, M. G.—(1961) 'A Course in Multivariate Analysis', London (Charles Griffin).
McGill, W. J.—(1954) *Psychometrika*, **19**, 97.
Silverstone, H., Campbell, C. B., Hosking, C. S., Lang, L. P. and Richardson, R. G.—(1963) *Med. J. Aust.*, **1**, 312.
Silverstone, H. and Gordon, D.—(1966) *Med. J. Aust.*, **2**, 733.

### APPENDIX

### *Information*

A system consisting of $N$ items divided into $k$ fixed categories is said to have " information content "

$$H = -\sum_{i=1}^{k} p_i \ln p_i$$

where $p_i$ = the proportion of items falling into the $i$th category and $\ln p_i$ = the natural logarithm of $p_i$.

$H$ is a maximum when the $p_i$ are all equal.

If the observations have been stratified into categories according to some quantity or quality denoted by $x$, the information content is denoted by $H(x)$. A two-way table of frequencies where stratification takes place simultaneously with respect to two variables, $x$ and $y$, has information content

$$H(x, y) = -\sum_i \sum_j p_{ij} \ln p_{ij}$$

where $p_{ij}$ is the proportion of items found in category $i$ for $x$ and category $j$ for $y$.

The information "shared by $x$ and $y$" or, alternatively, the information "transmitted from $x$ to $y$" or the "information on $y$ provided by $x$" is defined as

$$T(x; y) = H(x) + H(y) - H(x, y)$$

In the expression for $T(x; y)$ one may replace the variable $x$, usually regarded as the "independent" variable, by a set, or vector, of variables $x_1$, $x_2$, . . . etc., with consequent extension of the notation to $T(x_1, x_2, \ldots ; y)$.

The "dependent" variable $y$ in the present analysis has 2 values or levels only, namely "positive" or "negative" for the disease.

Conditional, or partial, information is defined (for example, for 3 independent variables) by expressions such as

$$T_{x_1}(x_2; y) = T(x_1, x_2; y) - T(x_1; y)$$

that is, the information on $y$ provided by $x_2$ when $x_1$ is fixed or given, is the difference between the information jointly provided by $x_1$ and $x_2$ and the information provided by $x_1$ alone. Similarly,

$$T_{x_1, x_2}(x_3; y) = T(x_1, x_2, x_3; y) - T(x_1, x_2; y)$$

and so on.

The fundamental theorem used in "stepwise" analysis is, illustrating again the case for 3 independent variables:

$$T(x_1, x_2, x_3; y) = T(x_1; y) + T_{x_1}(x_2; y) + T_{x_1, x_2}(x_3; y)$$

a theorem which generalizes to any number of independent variables.

For a good discussion of multivariate information concepts see McGill (1954) and Garner and McGill (1956).

The "$T$" measures have an important statistical property, namely that the distribution of the quantity $2NT$ in random sampling behaves like the distribution of "chi-square", so that under suitable safeguards as to sample size and with the appropriate determination of the "degrees of freedom" the significance of a value of $T$ may be determined by reference to ordinary chi-square tables.

The addition of a new independent variable in the stepwise procedure is equivalent to a further stratification within each of the sub-classes already created by earlier stratifications, and thus increases the number of categories by $s(k - 1)$ where $s$ = previous number of sub-groups and $k$ = number of levels of the new factor. To give an acceptable analogy with the "Regression" method (see below) in the construction of Fig. 6, the gain in information at any step was divided by the appropriate value of $s(k - 1)$.

### *Multiple Linear Regression and Discriminant Functions*

This topic is well presented in a number of texts (for example Kendall, 1961).

The stepwise regression procedure first labels as " $x_1$ " that $x$-variable which has the highest sum of squares for regression on $y$. It next examines all possible pairs $x_1$, $x_i$ ($i \neq 1$) and labels as " $x_2$ " that variable which produces the greatest increase in the sum of squares for the double regression on $y$. It then examines all possible triples $x_1$, $x_2$, $x_j$ ($j \neq 1$ or 2) to find $x_3$; and so on. The procedure may be stopped at any stage, preferably when a sufficiently large proportion of the total available sum of squares for regression has been exhausted. Library programs are found at any computer centre.

The gain in information at any stage is the increase in the regression sum of squares over that for the previous stage. No scale factor is required for different stages as was the case in the " Information " approach to Fig. 6, since each successive stage involves one degree of freedom only.

Kendall (1961) shows how the calculation of a " linear discriminant " function is formally equivalent to " coding " a " positive " as say $y = 1$ and a " negative " as $y = 0$ and finding the regression function of $y$ on the $x$-variables. Thus the technique and the computer programs designed for stepwise regression analysis can be used for stepwise discrimination analysis.

### *Smoothing Technique*

In calculating the smoothed values of $p_+$ for the construction of Fig. 7, 8 and 9 and Table IV attention was paid to the fact that data involving proportions usually require a linearizing transformation if they are to be fitted by a hyperplane. The transformation used here was to replace $p_+$ by $\ln[-\ln(1 - p_+)]$ with an appropriate weighting system (Fisher and Yates, 1963). The inverse transformation was then used to recover the smoothed values of $p_+$ illustrated as percentages in the figures and table listed above. In all cases the transformation led to a closer fit to the data.

References arising from the Appendix have already been given at the end of the main presentation.